# Geospatial distribution of *Mycobacterium tuberculosis* genotypes in Africa

**DOI:** 10.1371/journal.pone.0200632

**Published:** 2018-08-01

**Authors:** Violet N. Chihota, Antoinette Niehaus, Elizabeth M. Streicher, Xia Wang, Samantha L. Sampson, Peter Mason, Gunilla Källenius, Sayoki G. Mfinanga, Marnomorney Pillay, Marisa Klopper, Webster Kasongo, Marcel A. Behr, Nicolaas C. Gey van Pittius, Paul D. van Helden, David Couvin, Nalin Rastogi, Robin M. Warren

**Affiliations:** 1 DST/NRF Centre of Excellence for Biomedical Tuberculosis Research /SAMRC Centre for Tuberculosis Research, Division of Molecular Biology and Human Genetics, Faculty of Medicine and Health Sciences, Stellenbosch University, Tygerberg, South Africa; 2 The Aurum Institute, Johannesburg, South Africa; 3 School of Public Health, Faculty of Health Sciences, University of the Witwatersrand, Johannesburg, South Africa; 4 Department of Mathematical Sciences, University of Cincinnati, Cincinnati, Ohio, United States of America; 5 Biomedical Research and Training Institute, Harare, Zimbabwe; 6 Department of Clinical Science and Education, Södersjukhuset, Karolinska Institutet, Stockholm, Sweden; 7 National Institute for Medical Research Muhimbili Medical Research Centre, Dar es Saalam, Tanzania; 8 Department of Medical Microbiology University of KwaZulu Natal, Durban, South Africa; 9 Tropical Diseases Research Centre, Ndola, Zambia; 10 Division of Infectious Diseases, Department of Medicine McGill University Health Centre, Montreal, Quebec, Canada; 11 WHO Supranational TB Reference Laboratory, Institut Pasteur de la Guadeloupe, Abymes, Guadeloupe, France; Universidade Nova de Lisboa Instituto de Higiene e Medicina Tropical, PORTUGAL

## Abstract

**Objective:**

To investigate the distribution of *Mycobacterium tuberculosis* genotypes across Africa.

**Methods:**

The SITVIT2 global repository and PUBMED were searched for spoligotype and published genotype data respectively, of *M*. *tuberculosis* from Africa. *M*. *tuberculosis* lineages in Africa were described and compared across regions and with those from 7 European and 6 South-Asian countries. Further analysis of the major lineages and sub-lineages using Principal Component analysis (PCA) and hierarchical cluster analysis were done to describe clustering by geographical regions. Evolutionary relationships were assessed using phylogenetic tree analysis.

**Results:**

A total of 14727 isolates from 35 African countries were included in the analysis and of these 13607 were assigned to one of 10 major lineages, whilst 1120 were unknown. There were differences in geographical distribution of major lineages and their sub-lineages with regional clustering. Southern African countries were grouped based on high prevalence of LAM11-ZWE strains; strains which have an origin in Portugal. The grouping of North African countries was due to the high percentage of LAM9 strains, which have an origin in the Eastern Mediterranean region. East African countries were grouped based on Central Asian (CAS) and East-African Indian (EAI) strain lineage possibly reflecting historic sea trade with Asia, while West African Countries were grouped based on Cameroon lineage of unknown origin. A high percentage of the Haarlem lineage isolates were observed in the Central African Republic, Guinea, Gambia and Tunisia, however, a mixed distribution prevented close clustering.

**Conclusions:**

This study highlighted that the TB epidemic in Africa is driven by regional epidemics characterized by genetically distinct lineages of *M*. *tuberculosis*. *M*. *tuberculosis* in these regions may have been introduced from either Europe or Asia and has spread through pastoralism, mining and war. The vast array of genotypes and their associated phenotypes should be considered when designing future vaccines, diagnostics and anti-TB drugs.

## Introduction

The development and application of genotyping tools for *Mycobacterium tuberculosis* has greatly enhanced our understanding of the epidemiology of tuberculosis (TB) on a local [1] and global scale [2–5]. Three internationally standardized genotyping methods, IS*6110* DNA fingerprinting [6], Mycobacterial Interspersed Repetitive Unit-Variable Number of Tandem Repeat (MIRU-VNTR) typing [7] and spoligotyping [8] have been used extensively to quantify transmission [9], describe genetic diversity [3], determine the epidemiology of drug resistance [10–12] and identify mixed infections [13,14]. Spoligotyping data represents the largest body of genotyping data which has been formally organized into a global repository termed SpolDB [2–4,15]. This database has been through a number of reiterations and has recently been expanded to include MIRU-VNTR data, and is now called SITVIT [16]. Within this database clinical isolates have been grouped into distinct lineages such as Beijing, Central Asian (CAS), East-African Indian (EAI), Cameroon, Haarlem (H), Latin American Mediterranean (LAM), T, S, and X according to defined spoligotype signatures [17,18].

In 2002, Filliol *et al* used spoligotype data from SpolDB to present the first view of the global phylogeny of *M*. *tuberculosis* [2]. Subsequent studies described the population structure of *M*. *tuberculosis* complex (MTBC) on the different continents [4,19]. Findings from these studies are largely concordant with those from studies using the Long Sequence Polymorphism (LSP) to describe the global phylogeny of the 6 LSP lineages [5,20]. In Africa, the Euro-American lineage was found to be dominant. The CAS and EAI lineages were confined to East Africa [21], the East Asian lineage (i.e. Beijing) was predominantly found in Southern Africa and the *M*. *africanum* lineages were limited to West Africa and show significant geographical variation [22]. In 2013, isolates representing a seventh lineage were identified in Ethiopia [23]. Phylogenetic analysis of whole genome sequence data from clinical isolates representative of the 7 different LSP lineages using Bayesian and Maximum Parsimony methods predicted that the common ancestor of MTBC originated in Africa [24]. That study also showed co-evolution between host and pathogen and suggested that the pathogen was carried out of Africa by hunter-gatherers. Similar hypotheses have been proposed by others [20,25,26].

Recent studies have proposed the “back to Africa” hypothesis whereby *M*. *tuberculosis* was reintroduced into Africa as a consequence of trade and colonization [19,20]. The consequence of this reintroduction on the phylogeographical population structure of *M*. *tuberculosis* remains unknown. This study aimed to comprehensively describe the population structure of *M*. *tuberculosis* isolates in Africa using data from the SITVIT2 database and literature to explore geospatial strain diversity and expand our limited knowledge on regional differences.

## Materials and method

### Data collection and access on SITVIT

Spoligotyping data for isolates originating from African countries were extracted from the SITVIT2 database [27,28]. In addition, spoligotype data for isolates from Europe and Western, South and South Eastern Asia were extracted from SITVITWEB (www.pasteur-guadeloupe.fr:8081/SITVIT_ONLINE/). Isolates were excluded if they were from non-human hosts, were atypical mycobacteria or members of the MTBC other than *M*. *tuberculosis*. All spoligotyping signatures that were not yet associated to a well-defined genotypic lineage in the SITVIT2 database were designated as “Unknown”.

### Data collection from literature

PubMed was searched using the terms “Tuberculosis AND Spoligo* AND country name” to identify manuscripts reporting spoligotype data for 11 African countries which have not submitted data to SITVIT2 [22,29–39]. To avoid duplication, data from countries which had deposited their spoligotyping data in SITVIT was not included. Only spoligotype data for *M*. *tuberculosis sensu stricto* isolates were extracted.

### Ethics statement

The data included in this study is anonymized and freely available from SITVITWEB and the cited literature.

### Geographical distribution

To gain a broad overview of the African *M*. *tuberculosis* population structure, isolates were assigned to major lineages. The distribution of genotypes was described by country of origin. A map of Africa was prepared showing the proportion of isolates belonging to the respective spoligotype lineages for the respective countries (3 letter country codes according to http://en.wikipedia.org/wiki/ISO_3166-1_alpha-3). Country specific spoligotyping data was included in the analysis if spoligotype data for ≥ 100 isolates was available. Isolates belonging to the Turkey and Ural (U) lineages were excluded owing to their limited frequency in Africa. In addition, maps were prepared to show the proportion of *M*. *tuberculosis* isolates belonging to the different sub-lineages of the respective major spoligotype lineages if the major lineage constituted ≥ 15% of the *M*. *tuberculosis* isolates for that country.

### Principal component analysis

Given the complex nature of the data, a principal component analysis (PCA) is an appropriate mathematical tool to reveal underlying patterns within the data. This analysis was completed using R (version 3.2.0) [40] and visualized using the ggbiplot R package [41]. PCA analysis of the geographical distribution of the major lineages in countries belonging to Africa, Europe and Western, South and South East Asia was done using the proportions of the different lineages and not the spoligotype itself. This analysis included spoligotyping data for the Beijing, Cameroon, CAS, EAI, H, LAM, Manu, and S lineages. Spoligotype data for the T lineage was excluded from the PCA analysis, since these isolates were present in most countries included in the analysis. In addition, data for the X lineage (based on the small proportion of strains in this group) and isolates with unassigned spoligotypes were excluded. Independent PCA analyses were done to determine the distribution of the isolates belonging to the respective sub-lineages of the major lineages LAM and T. PCA analysis was not done for the Beijing, EAI, Manu, S, and X lineages owing to the limited number of countries where these strains were present in sufficient proportion.

### Hierarchical cluster analysis

To confirm the clustering, we used R function pvclust [42], which performs hierarchical cluster analysis via function hclust and automatically computes p-values for all clusters contained in the clustering of original data. The AU p-value represents the "approximately unbiased" p-value, which is calculated by multiscale bootstrap resampling and is a value between 0 and 1. The clusters (edges labeled in grey) with high AU values (e.g. 95%) can be considered as strongly supported by data. As the estimation of the AU p-values also has uncertainty, 100,000 bootstraps were run in order to decrease the standard error. The clusters with AU greater than 95% are highlighted with red rectangles.

In order to determine whether a relationship in the proportion of major lineage existed between the African, European and Asian countries, PCA and pvclust data for European and Western, South and South East Asian countries were analysed.

### Phylogenetic tree analysis

BioNumerics software version 6.6 (Applied Maths, Sint-Martens-Latem, Belgium; available at the following link: http://www.applied-maths.com/bionumerics) was used to highlight evolutionary relationships between main spoligotypes present in Africa. Minimum spanning trees were drawn based on spoligotyping patterns having a SIT number, and belonging to the following lineages: LAM, T, H, Beijing, CAS, X, Cameroon, EAI, S, and Manu. Minimum Spanning Trees are undirected graphs in which all samples are connected together with the fewest possible connections between nearest neighbors.

## Results

### Overview of *M*. *tuberculosis* genotypes in Africa

A total of 112,683 mycobacterial isolates in the SITVIT2 database were screened for eligibility. All isolates from non-African countries (n = 99,196), non-human hosts (n = 965), members of the MTBC other than *M*. *tuberculosis* (n = 598), and atypical mycobacteria (n = 41) were excluded, leaving 11,883 *M*. *tuberculosis* isolates. These isolates represented spoligotype data from individual patients in 25 countries in the Africa region (S1 Table). Review of the literature added isolates from an additional 11 countries resulting in a total of 15522 *M*. *tuberculosis* isolates with spoligotype data in Africa (S1 Table). African countries not represented included Botswana, Burundi, Cabo Verde, Chad, Congo, Republic of the Congo, Equatorial Guinea, Eritrea, Gabon, Lesotho, Liberia, Mauritania, Niger, Sao Tome and Principe, Seychelles, Somalia, South Sudan, Swaziland, and Togo.

A further eleven countries, namely Angola, Benin, Comoros, Kenya, Libya, Mali, Mauritius, Namibia, Reunion, Senegal, and Sierra Leone were excluded because each country contributed ≤100 *M*. *tuberculosis* isolates (n = 685) (S1 and S2 Tables). In addition, 38 isolates from the Turkey lineage previously designated as LAM 7, and 72 isolates from the U lineage were also excluded. A total of 14727 isolates were included in the analyses.

### Major *M*. *tuberculosis* genotypes identified in regions of Africa

13607 isolates (92.4%) were assigned to one of 10 major spoligotype lineages, while the remaining 1120 (7.6%) isolates could not be assigned to a lineage and were grouped as “Unknown”. The six most frequent lineages were T (24.8%), LAM (19.3%), Cameroon (11.4%), H (9.0%), Beijing (7.4%), and CAS (6.4%). These six lineages accounted for 78.3% of all isolates from the Africa region (S1 Table).

### Phylogeographical clustering of major *M*. *tuberculosis* lineages in African countries

**Fig 1:**
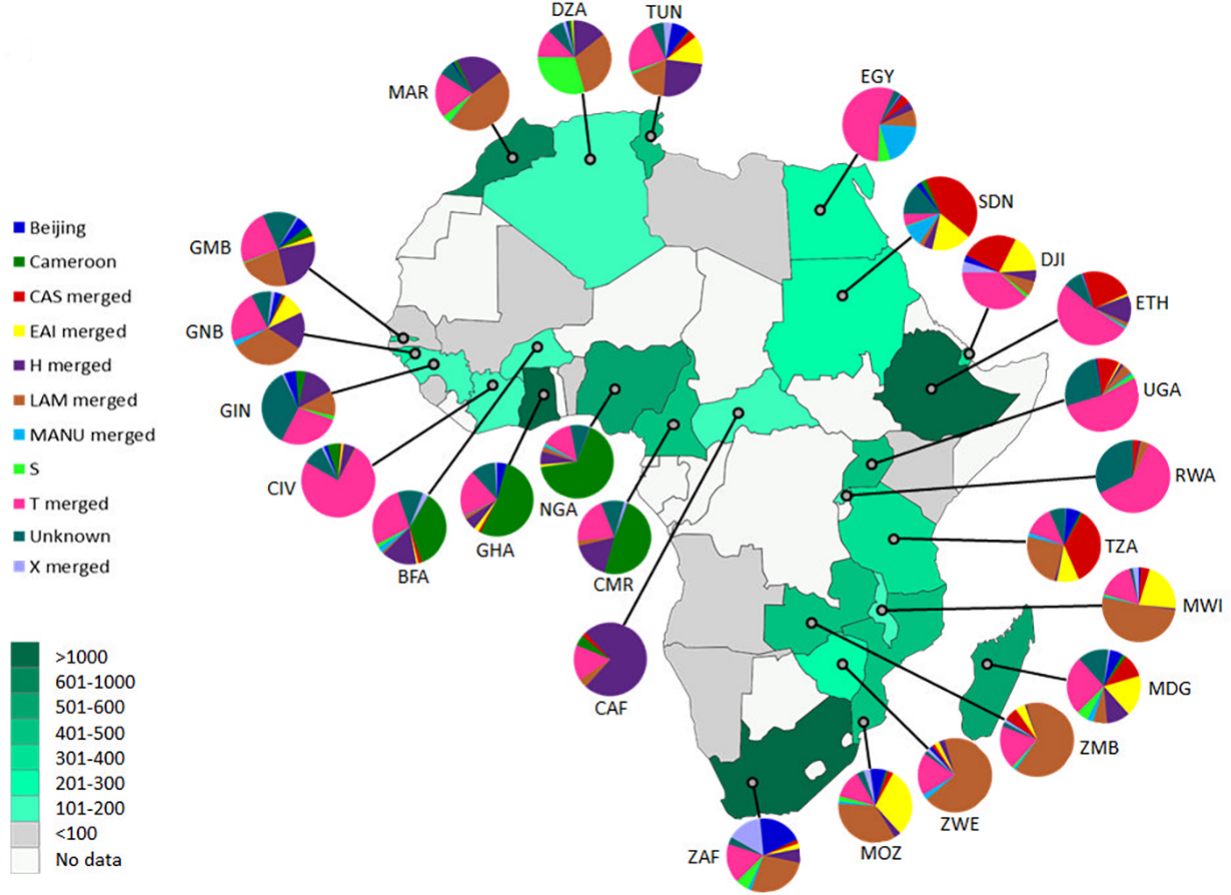
Geospatial distribution of *M*. *tuberculosis* lineages in Africa. Each pie chart segment reflects the relative proportion of *M*. *tuberculosis* isolates belonging to respective major lineages for each country (see colour chart for the respective major lineages). Each country has been shaded according to the number of isolates contributed to the analysis (see colour intensity chart). Country codes (http://www.worldatlas.com/aatlas/ctycodes.htm).

**Fig 2:**
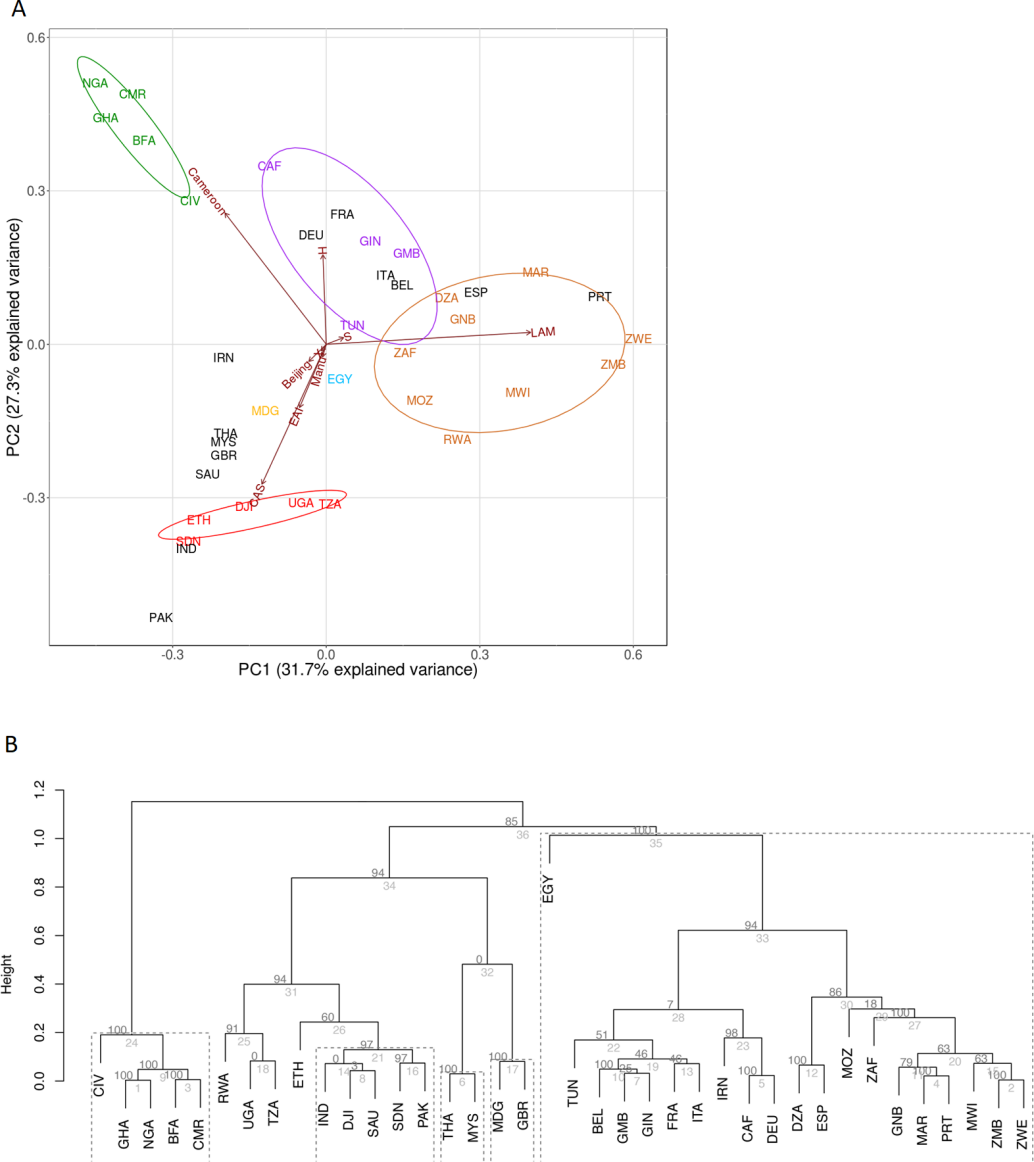
Clustering of countries according the proportion of *M*. *tuberculosis* isolates present in a specific lineage. Only data from the Beijing, Cameroon, CAS, EAI, H, LAM, Manu, and S lineages was included. Country codes according to (http://www.worldatlas.com/aatlas/ctycodes.htm). (A) Principle component analysis: African countries in the PCA plot are coloured based on their most dominant lineage: CAS (red), Cameroon (green), H (purple), LAM (brown), Manu (blue), and EAI (yellow). European and Asian countries are shown in black. Overlapping country codes in the PCA plot indicate a similar distribution of *M*. *tuberculosis* lineages in the respective countries. (B) pvclust analysis: The clusters edges are numbered in grey and the AU p-values are shown in black. Strongly supported clusters with AU greater than 95% are highlighted with a dotted line.

We assessed the intra-country lineage proportions in 25 African countries for which data for >100 isolates was available. Fig 1 shows the proportion of isolates representative of the 10 different *M*. *tuberculosis* lineages in these 25 countries. PCA and hierarchical cluster analysis using data from the 8 dominant lineages; Beijing, Cameroon, CAS, EAI, H, LAM, Manu, and S, showed a strong correlation with the groupings of countries by geographical location (Fig 2A and 2B). From the PCA analysis, principal component 1, which explained 31.7% of the variance in the data separated out countries based on either Cameroon/CAS versus LAM dominance. Principal component 2, which explained a further 27.3% of the variance in the data, further divided countries by Cameroon lineage compared to CAS dominance. Pvclust analysis showed similar results which largely correspond to the clustering identified in the PCA analysis. Southern African countries (South Africa, Mozambique, Zimbabwe, Zambia, and Malawi) grouped loosely together with Northern and Western African countries (Algeria, Morocco and Guinea Bissau) (AU value 86%) based on the high percentage of LAM in these regions. East African countries (Ethiopia, Sudan, Djibouti, Uganda, and Tanzania) (AU value 94%) showed a grouping based on the prevalence of isolates belonging to the CAS lineage. A dominance of the Cameroon lineage was seen in Central Africa with Nigeria, Cameroon, Ghana, Bukia Faso, and Cote Ivoire grouping together (AU value >95%). Countries which had a high percentage of isolates classified as belonging to the H lineage (Central African Republic, Guinea, Gambia, and Tunisia) showed a loose grouping together, however these countries generally showed a mixed distribution of lineages, with an influence of LAM lineages preventing a close clustering. Egypt showed a uniquely dominant Manu lineage and therefore did not cluster closely with other Northern African countries. Similarly, Madagascar showed a higher proportion of EAI than other African countries and therefore did not belong to a cluster. Countries Tunisia, South Africa, Egypt, and Madagascar have the highest lineage diversity and were therefore positioned towards the center of the PCA plot.

### Phylogeographical clustering of major *M*. *tuberculosis* sub-lineages in African countries

In order to describe the *M*. *tuberculosis* population structure in finer detail, the proportion of isolates representing the sub-lineages of each major lineage (CAS, EAI, LAM, H and T) were plotted onto their country of origin if they contributed ≥ 15% of the isolates causing disease in the respective country, as assessed by the present dataset (Figs 3, 4, 5 and 6 and S1–S6 Figs, and S2 Table).

**Fig 3:**
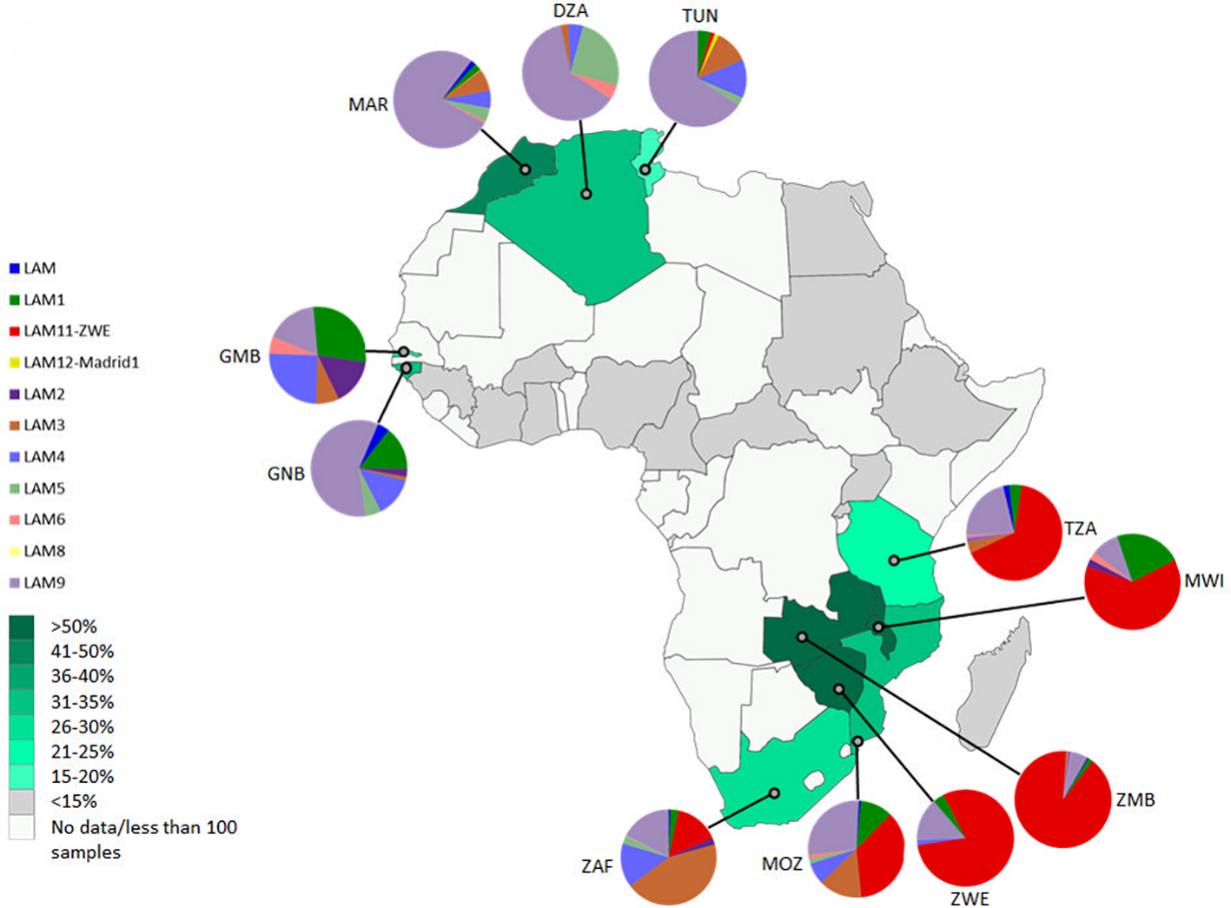
Geospatial distribution of *M*. *tuberculosis* isolates belonging to the LAM sub-lineage. Country specific spoligotype data was only included if the country had >100 *M*. *tuberculosis* isolates and ≥15% of these isolates were from the LAM lineage. The sizes of the pie chart segments depict the proportion of isolates belonging to the different LAM sub-lineages (see colour chart for the respective sub-lineages). Each country has been shaded according to the proportion of LAM lineages isolates present in that country (see colour intensity chart). Country codes (http://www.worldatlas.com/aatlas/ctycodes.htm).

**Fig 4:**
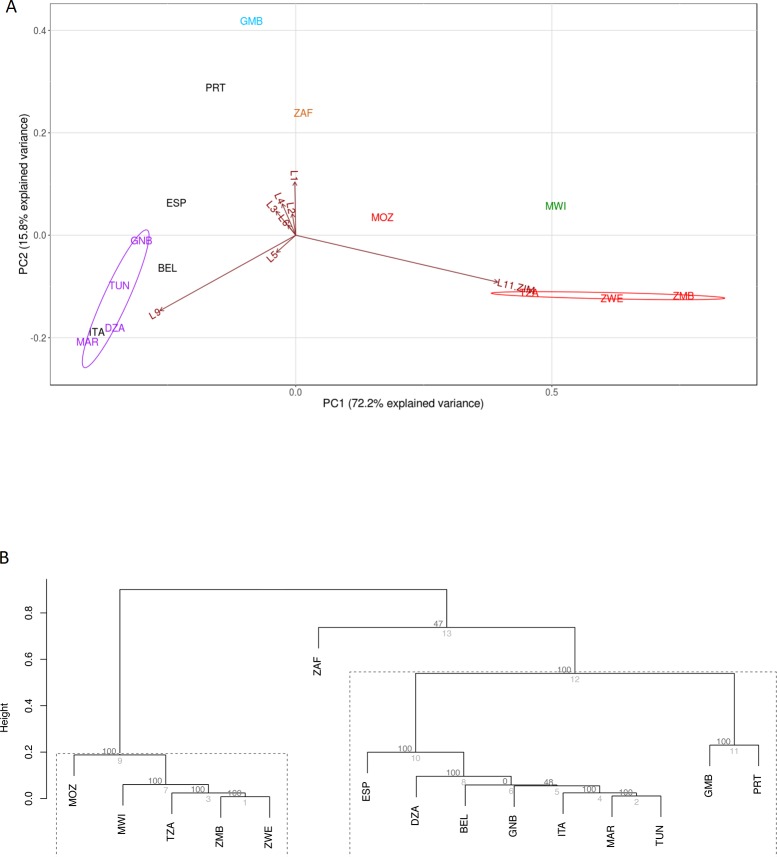
Clustering oof countries according the proportion of *M*. *tuberculosis* isolates belonging to different LAM sub-lineages. (A) Principle component analysis: African countries in the PCA plot are coloured based on their most dominant LAM sub-lineage: LAM1 (blue), LAM3 (blown), LAM9 (purple), LAM11-ZIM (red). PCA plot axes have been labelled with an “L” to indicate LAM followed by the sub-lineage number. European and Asian countries are shown in black. Overlapping country codes in the PCA plot (Morocco and Italy, Tunisia and France) indicate a similar distribution of LAM sub-lineages in the respective countries. (B) pvclust analysis: The clusters edges are numbered in grey and the AU p-values are shown in black. Strongly supported clusters with AU greater than 95% are highlighted with a dotted line. Country codes (http://www.worldatlas.com/aatlas/ctycodes.htm).

**Fig 5:**
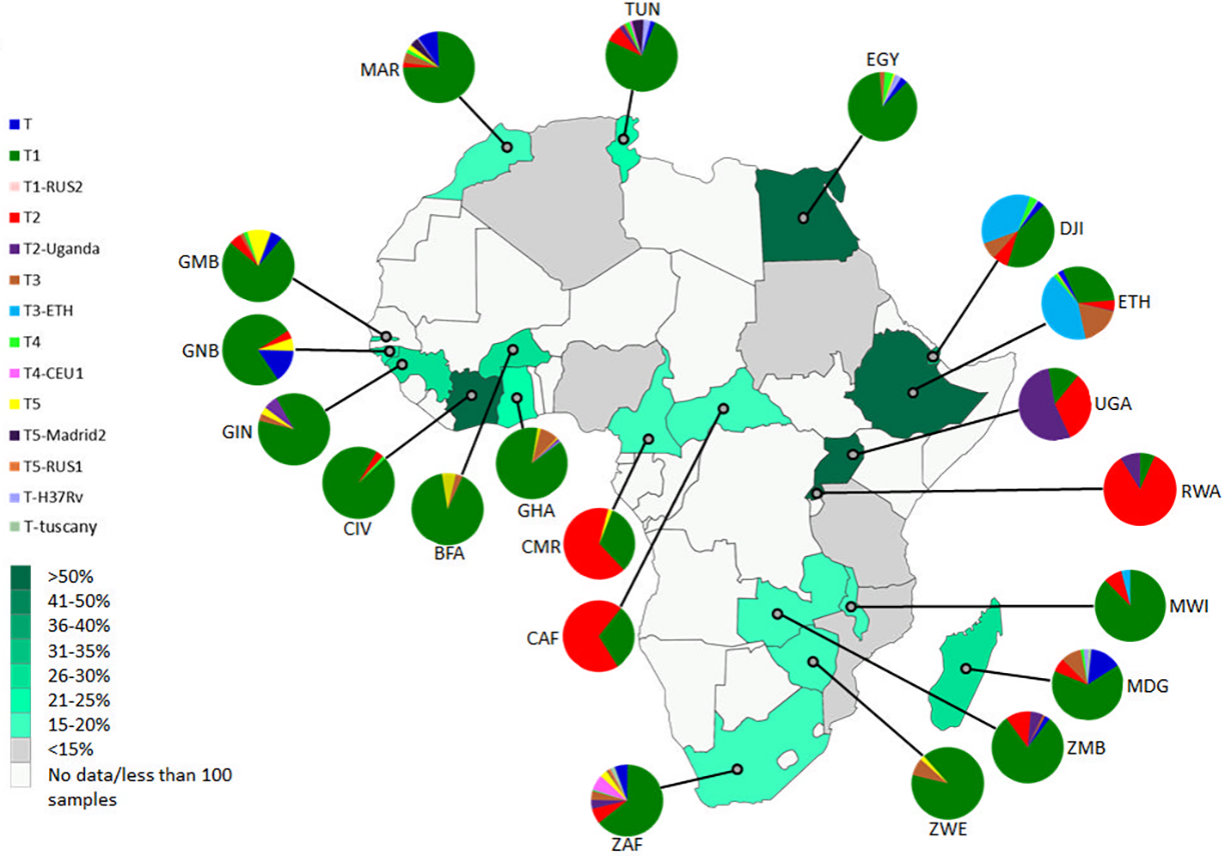
Geospatial distribution of *M*. *tuberculosis* isolates belonging to the T sub-lineages. Country specific spoligotype data was only included if the country had >100 *M*. *tuberculosis* isolates and ≥15% of these isolates were from the T lineage. The sizes of the pie chart segments depict the proportion of isolates belonging to the different T sub-lineages (see colour chart for the respective sub-lineages). Each country has been shaded according to the proportion of T sub-lineages isolates present in that country (see colour intensity chart). Country codes (http://www.worldatlas.com/aatlas/ctycodes.htm).

**Fig 6:**
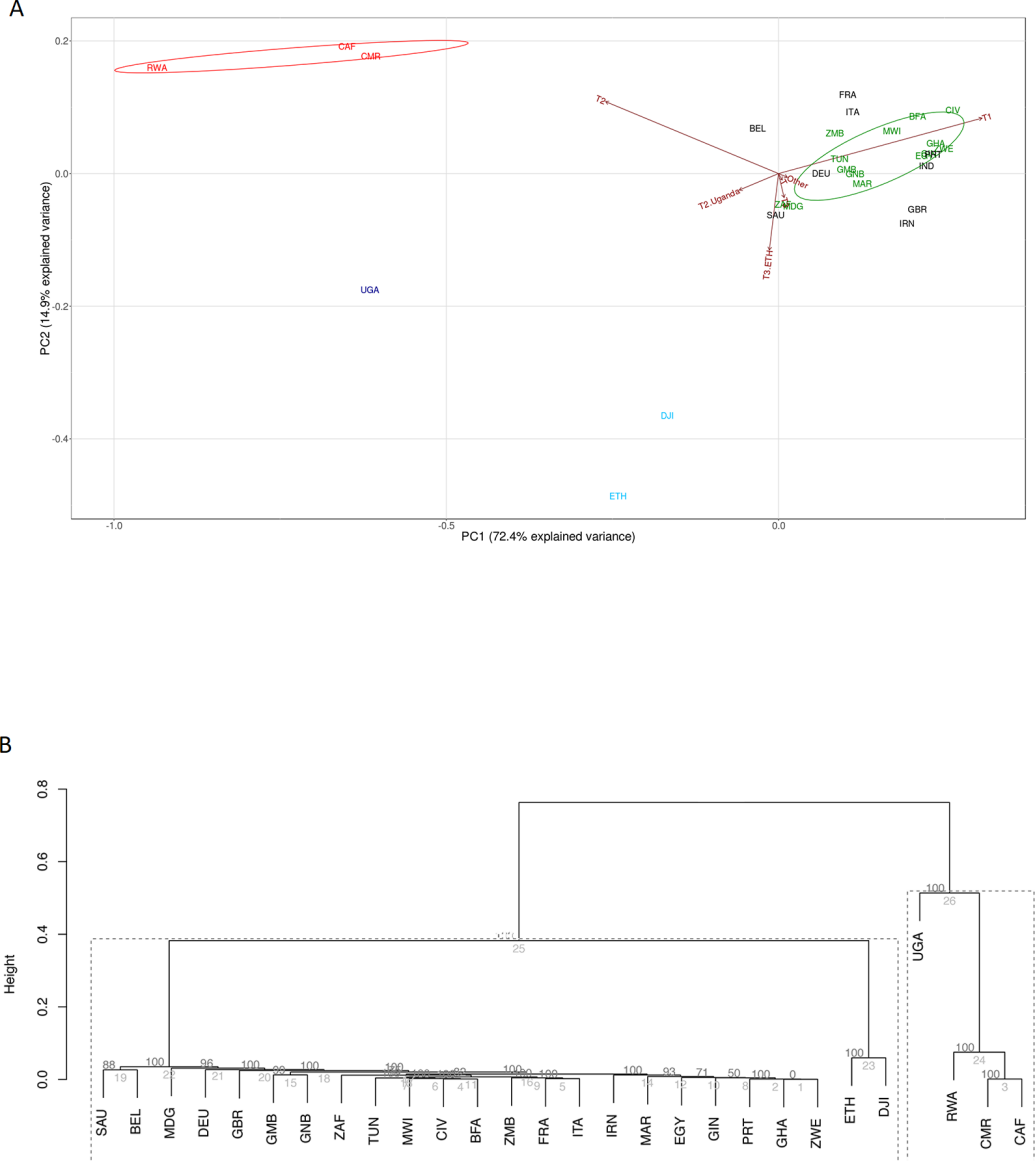
Clustering of countries according the proportion of *M*. *tuberculosis* isolates belonging to the T sub-lineages. (A) Principle component analysis: African countries in the PCA plot are coloured based on their most dominant T sub-lineage: T1 (green), T2 (red), T2-Uganda (purple), T3-Ethiopia (turquoise). European and Asian countries are shown in black. Overlapping country codes in the PCA plot (South Africa and Madagascar, Gambia and Guinea Bissau, Egypt and Guinea) indicate a similar distribution of T sub-lineages in the respective countries. (B) pvclust analysis: The clusters edges are numbered in grey and the AU p-values are shown in black. Strongly supported clusters with AU greater than 95% are highlighted with a dotted line. Country codes (http://www.worldatlas.com/aatlas/ctycodes.htm).

#### LAM sub-lineages

Fig 3 shows the distribution of *M*. *tuberculosis* isolates with the LAM genotype in African countries. A PCA analysis was done in order to determine the influence of LAM sub-lineage on geographical clustering. Principal component 1 of the PCR analysis (Fig 4A) explains the majority of the variance in the data (72.2%), and separates countries based on either a high LAM11-ZWE or LAM9 influence, which in turn reflects their geographical location (Fig 3). The pvclust analysis corresponds to the clustering identified in the PCA analysis (Fig 4B). Zambia, Zimbabwe, and Tanzania grouped together based on the high percentage of LAM11-ZWE in these countries and low percentage of other LAM subtypes (AU value >95%). Malawi was separated away from the main LAM11-ZWE cluster due to the high percentage of LAM1 isolates, despite the presence of a large proportion of LAM11-ZWE isolates. Guinea Bissau, Tunisia, Algeria, and Morocco grouped together based on the high percentage of LAM9 isolates in these countries and low percentage of other subtypes (AU value >95%). Gambia was separated from this grouping due to the high percentage of LAM1 and LAM4 (Fig 4A and 4B). Similarly, South Africa did not group with any of the other countries because of the high proportion of LAM3 isolates. Fig 4A and 4B also includes LAM sub-lineage data from Portugal, Spain, Belgium and Italy. Spain, Belgium, and Italy are all dominated by LAM9, and cluster closer with Northern African countries (such as Algeria, Morocco, Tunisia, and Guinea Bissau), while Portugal and Gambia have a larger percentage of the LAM1 component and therefore separates away from the LAM9 cluster (AU value >95%).

#### T lineages

The geospatial distribution of the T2 sub-lineages and the corresponding PCA and pvclust analyses are shown in Fig 6. Principal component 1 explains the majority of the variance in the data (72.3%), and separates countries that are predominantly T1 dominated from countries with high percentages of T2, T2-Uganda, or T3-Ethiopia (Fig 6A). The T1 subtype is dominant in 14 out of the 20 (70%) African countries and exhibit a strong clustering in the PCA and pvclust analyses (AU value >95%) (Fig 6A and 6B). Cameroon and Central African Republic which have a high percentage of T2 formed a separate cluster with Rwanda (AU value >95%). As expected, Uganda has a high proportion of T2-Uganda. Djibouti and Ethiopia have high proportions of T3-Ethiopia. The European (France, Great Britain, Germany, Belgium and Italy) and South Asian countries (Iran and Saudi Arabia) cluster with other T1 dominated African countries.

#### CAS lineage

Isolates belonging to the CAS lineage were observed in Eastern Africa. The geospatial distribution of CAS sub-lineages is shown in S1 Fig (representing countries having >15% CAS isolates). Pvclust grouped Saudi Arabia, Pakistan, India, Sudan, Ethiopia (AU value >95%) (S2 Fig).

#### EAI lineages

S3 Fig shows the distribution of EAI sub-lineages for countries that have > 15% EAI isolates. Members of the EAI lineage were largely restricted to the East African countries Sudan (17.6%), Djibouti (16.6%), Malawi (21.6%), Madagascar (17.9%), and Mozambique (30.3%). The data showed three strong groupings; Djibouti and Madagascar (AU >95%), Malaysia, Sudan and Thailand (AU >95%) and Great Britain and India (AU>95%) (S4 Fig).

#### H lineage

S5 Fig shows the distribution of H sub-lineages in Africa, with the most predominant sub-lineages in Africa being H1 and H3. A small proportion of sub-lineage H2 isolates were identified in Northern Africa. Pvclust analysis strongly supported grouping Gambia and Central African Republic, Cameroon and Iran, Tunisia, Spain and Belgium, and Morocco, Italy, France and Germany (S6 Fig)

#### Beijing lineage

Members of the Beijing lineage were found to be over-represented in South Africa accounting for 19.2% of all TB cases (Fig 1). Isolates belonging to this lineage were seen to a lesser extent in other countries: Mozambique (6.9%), Madagascar (5.5%) and Tanzania (6.4%) from the Southern African region; Guinea (5.3%) and Gambia (5.2%) from the West African region; and Tunisia (7.7%) in North Africa.

#### MANU lineage

Members of the ancient Manu lineage were over-represented in Egypt (19.1%) and Sudan (9.4%) relative to other countries in Africa.

#### X lineages

Isolates belonging to the X family were over represented in South Africa (15.3%) as well as Ghana (7.7%)

#### S lineage

Isolates belonging to the S lineage were most frequently observed in Algeria (29.7%) and to a lesser extent in South Africa (5.8%), Madagascar (5.1%) and Egypt (5.5%).

#### Clustering of *M*. *tuberculosis* lineages cultured in Africa, Europe and Asia

From Fig 2A and 2B it is evident that Spain and Portugal grouped with countries with LAM dominance. France, Germany, Italy, and Belgium fell into the loosely grouped H dominant cluster. The unique distribution of EAI, X and CAS in the Great Britain caused a separation from other European countries and grouped most closely Madagascar where the EAI lineage was dominant. Asian countries were dominated by CAS, EAI, H and Beijing strains. Pakistan, India and Saudi Arabia cluster most closely with the CAS dominant Eastern African countries (AU value 94%). India and Saudi Arabia however have quite a mixed distribution with a large influence of the EAI strain. Although Iran (Iran) also shows a large proportion of CAS, it is dominated by the H strain and clustered with Central African Republic and Germany (AU value 98%). Iran does not cluster with any of the other African nor Asian countries. Malaysia and Thailand both have a high distribution of both EAI and Beijing forming a close cluster (AU value >95%).

#### Minimum spanning tree analysis

The Minimum Spanning Tree based on all available spoligotype international types (SITs) (S7 Fig) highlighted evolutionary relationships and distance between SITs belonging to LAM, T, H, Beijing, CAS, X, Cameroon, EAI, S, and Manu. All represented lineages were well organized with isolates belonging to T lineage appearing at the central position and isolates belonging to T3-Ethiopia sub-lineage (particularly SIT149), were distinguishable from other T lineage isolates. Isolates belonging to the Cameroon lineage (particularly defined by SIT61) were located between T (represented by SIT44/T5) and LAM (represented by SIT42/LAM9) lineages, but closer to T patterns, suggesting the unique feature of the Cameroon group. Isolates belonging to Manu lineage (better represented by SIT54/Manu2) were concentrated between T (represented by SIT53/T1) and EAI (represented by SIT48/EAI1-SOM). Isolates belonging to EAI lineage were distributed partially between Manu and CAS lineages, with CAS being located in the upper position of the Minimum Spanning Tree. Isolates belonging to Beijing lineage were visible on the lower part of the tree.

Minimum Spanning Trees drawn focusing on LAM S8A Fig and T (S8B Fig) lineages showed a more accurate view of organization of sub-lineages and SITs belonging to these lineages. The Minimum Spanning Tree based on LAM lineage spoligotypes (S8A Fig) displayed patterns belonging to LAM3 sub-lineage, essentially represented by SIT33, at the upper part of the tree, whereas isolates belonging to LAM11-ZWE sub-lineage (mainly represented by SIT59) were located at the lower position. The latter sub-lineage was differentiated from SIT42/LAM9 by the spoligotype pattern SIT64/LAM6 and another SIT less represented.

The Minimum Spanning Tree based on T lineage spoligotypes (S8B Fig) also highlighted a visible separation between SIT53/T1 (at the central position) and SIT149/T3-Ethiopia (appearing at the lower position). Isolates belonging to T1 sub-lineage were rather scattered throughout the tree. Also noticeable is the exclusion of patterns SIT1737/T-Tuscany and SIT254/T5-RUS1 appearing on the right upper corner of the tree. As might be expected, patterns belonging to T2-Uganda were following patterns belonging to T2 sub-lineage. However, a group of T1 sub-lineage isolates (represented by SIT244) was also following the group of T2 sub-lineage isolates. Classification of this profile may be unclear.

## Discussion

This is the first study to comprehensively describe the population structure of *M*. *tuberculosis* on a country, regional and continental scale. All of the major spoligotype lineages were found to be present in Africa. However, there were clear and distinct differences in the geographical distribution of the major lineages with regional clustering. This may reflect a founder effect where certain *M*. *tuberculosis* strains were initially introduced into defined areas as a result of colonization and sea trade [19,20] and later became distributed over a larger area as a consequence of movement of individuals. The introduction of CAS and EAI lineage strains into East Africa probably reflects the historic Indian Ocean trade route, which stretched between Madagascar in the South, Egypt in the North, and Western, South and South East Asia. This is supported by the over-representation of the CAS-Delhi sub-lineage in Saudi Arabia, Iran, Pakistan and India, and the EAI-5 sub-lineage in Saudi Arabia, India and Malaysia. The CAS-Delhi and EAI-5 sub-lineages are the possible progenitor strains to CAS-Kili and EAI-Madagascar and EAI-BDG, respectively, as they have the most intact direct repeat region. The CAS-Kili sub-lineage appears to have evolved in Tanzania and subsequently spread to neighboring countries, however, this lineage has not become dominant in those neighboring countries. It is not clear where the EAI-Madagascar sub-lineage evolved, although it is strongly associated with TB in Djibouti and Madagascar, possibly reflecting movement of people between these two countries, both of which were colonized by France.

The TB epidemic in Sothern Africa is dominated by the LAM11-ZWE sub-lineage which evolved from the LAM9 (RD174/RDRio) strain through expansion of the ETRB variable number tandem repeat and loss of spacers 27 to 30 in the direct repeat region [43]. The progenitor LAM9 (RD174/RDRio) strain is thought to have originated from Portugal, a country which lead numerous expeditions to Southern Africa and traded extensively in this region thereby explaining the introduction of this strain. The LAM11-ZWE strain is now distributed throughout Southern Central Africa possibly reflecting trade within the historical Federation of Rhodesia and Nyasaland and between neighboring Tanzania and Mozambique. The LAM9 strains in North Africa (Tunisia, Algeria, and Morocco) differ from those identified in Portugal probably reflecting trade with the Eastern Mediterranean region as these countries formed part of the Ottoman Empire. The LAM9 (RD174/RDRio) isolates from patients in Gambia differ from those found in North Africa and are largely characterized by the presence of RD174/RDRio, the predominant genotype identified in Portugal [43]. Portugal traded with Gambia and neighboring Guinea Bissau from the 15^th^ century and later colonized Guinea Bissau.

West Africa is dominated by the presence of the Cameroon lineage, present in Burkina Faso, Ghana, Nigeria and Cameroon. The large geographic distribution of this lineage reflects historic and continuing intra-regional movement which was further promoted with the establishment of the Economic Community of West African States in 1979. It is unknown whether this Cameroon lineage evolved in West Africa or whether it or a precursor was introduced during colonization. Interestingly, this lineage has been isolated in France and Belgium which may reflect migration from West Africa to Europe.

The origin of the T lineage in Africa remains largely unknown as this ill-defined lineage is present in high proportions in most African countries. Our analysis shows that the T2 sub-lineage is spread across the central region of Africa. Strains from this lineage potentially evolved into the T2-Uganda sub-lineage, in Uganda and spread to neighboring Rwanda. The T3 sub-lineage (defined by the loss of spoligotyping spacer 13) was largely found in Ethiopia and it is hypothesized that this lineage evolved into T3-Ethiopia strain through the loss of spoligotyping spacer 10–12 and 14–19. Strains of both the T3 and T3-Ethiopia sub-lineages were also identified in neighboring Djibouti and Saudi Arabia possibly reflecting modern day movement of Ethiopian refugees travelling to Saudi Arabia via Djibouti.

*M*. *tuberculosis* cultured from patients resident in South Africa showed the greatest diversity as well as the greatest abundance of Beijing lineages. Interestingly, the Beijing lineage strains found in Cape Town show similar genetic features to the Beijing strains from Southeast Asia [44,45], possibly reflecting the importation of slaves. The success of the Beijing lineage in South Africa has been ascribed to host pathogen compatibility and an association between HLA-B27 [46]. We could also speculate that one reason why some lineages are prevalent in specific regions/countries is that they might be well adapted to some populations [47]. The low proportion of Beijing lineage stains in other African countries situated on the East coast of Africa is surprising given the traditional trade routes between Africa and Asia.

In recent years the increasing interaction between people on a worldwide scale due to advances in technology and transportation will likely define new patterns of *M*. *tuberculosis* distribution. More specifically in Africa refugee migration, driven by conflict or economic hardships is very common. This could influence the population structure of *M*. *tuberculosis* given the success of strains such as Beijing or LAM. These strains may be taking over the traditional ones and in some areas may emerge as new strains, such as in the case of T family. However we do not have strong evidence to show that the population structures are changing and more longitudinal studies are needed. Patterns of distribution and percentages of newer lineages emerging in areas where they would not be traditionally expected may help generate hypothesis about the direction of the general epidemic in future, given new patterns of migration and globalization.

We acknowledge that this study has a number of limitations. First, our data was not substantiated with more robust analysis like MIRU-VNTR or whole genome sequencing. This could have increased the discriminatory power, thereby optimizing the classification of the *M*. *tuberculosis* strains. Second, the PCA was carried out using either lineages or sub-lineages, and not the SITs. Considering that some of the sub-lineages might be polyphyletic, corresponding strains between countries may not fully represent a true monophyletic branch, and in such cases a shared evolutionary history for the strains in question might have not occurred. Nevertheless, it would have been too cumbersome to perform PCA analysis of *M*. *tuberculosis* isolates based on thousands of SITs, with inherently complicated results and interpretations. We therefore chose to perform PCA using either lineages or sub-lineages for the time being. When the next database is released with a significantly greater number of strains and SITs worldwide in near future, and SITs from Africa are better characterized, it might be worthwhile to run PCA analysis of selected SITs. Third, the strain population structure in many of the countries was defined by a single study. This could introduce bias depending on how representative the study was. However, our observation of geographical clustering suggests that the data included largely reflects the strain diversity of that country. Fourth, data from a number of countries was not available. This together with the exclusion of countries with less than 100 isolates may have prevented the detection of new regional clustering. Fifth, it is not possible to determine whether the observed clustering of *M*. *tuberculosis* lineages or sub-lineages reflects recent or historic movement of people as spoligotyping was only implemented as a genotyping tool in 1997. This would have been more feasible by using MIRU-VNTR in addition to spoligotyping which would have allowed robust evaluation of clonal stability. Last, the sampling period for these studies was different and represented different time-points of ongoing epidemic, therefore we cannot exclude the possibility that clustering may be missed if the population structure of *M*. *tuberculosis* has changed.

In summary, this study suggests a more complex population structure than was previously reported using either spoligotyping [18] or LSP data [5]. Furthermore, this study highlighted that the TB epidemic in Africa is driven by regional epidemics characterized by genetically distinct lineages of *M*. *tuberculosis*. TB in these regions may have been introduced from either Europe or Asia and has spread through pastoralism, mining and war. The vast array of genotypes and their associated phenotypes should be considered when designing future vaccines, diagnostics and anti-TB drugs.

## Supporting information

S1 Fig Geospatial distribution of *M*. *tuberculosis* isolates belonging to the CAS sub-lineages. Country specific spoligotype data was only included if the country had >100 *M*. *tuberculosis* isolates and ≥15% of these isolates were from the CAS lineage. The sizes of the pie chart segments depict the proportion of isolates belonging to the different CAS sub-lineages (see colour chart for the respective sub-lineages). Each country has been shaded according to the proportion of CAS sub-lineages isolates present in that country (see colour intensity chart). Country codes (http://www.worldatlas.com/aatlas/ctycodes.htm).
(TIF)

S2 Fig pvclust analysis of *M*. *tuberculosis* isolates belonging to the CAS sub-lineages. The clusters edges are numbered in grey and the AU p-values are shown in black. Strongly supported clusters with AU greater than 95% are highlighted with red rectangle. Country codes (http://www.worldatlas.com/aatlas/ctycodes.htm).
(TIF)

S3 Fig Geospatial distribution of *M*. *tuberculosis* isolates belonging to the EAI sub-lineage. Country specific spoligotype data was only included if the country had >100 *M*. *tuberculosis* isolates and ≥15% of these isolates were from the EAI lineage. The sizes of the pie chart segments depict the proportion of isolates belonging to the different EAI sub-lineages (see colour chart for the respective sub-lineages). Each country has been shaded according to the proportion of EAI sub-lineages isolates present in that country (see colour intensity chart). Country codes (http://www.worldatlas.com/aatlas/ctycodes.htm).
(TIF)

S4 Fig pvclust analysis of *M*. *tuberculosis* isolates belonging to the EAI sub-lineages. The clusters edges are numbered in grey and the AU p-values are shown in black. Strongly supported clusters with AU greater than 95% are highlighted with red rectangle. Country codes (http://www.worldatlas.com/aatlas/ctycodes.htm).
(TIF)

S5 Fig Geospatial distribution of *M*. *tuberculosis* isolates belonging to the H sub-lineage. Country specific spoligotype data was only included if the country had >100 *M*. *tuberculosis* isolates and ≥15% of these isolates were from the H lineage. The sizes of the pie chart segments depict the proportion of isolates belonging to the different H sub-lineages (see colour chart for the respective sub-lineages). Each country has been shaded according to the proportion of H sub-lineages isolates present in that country (see colour intensity chart). Country codes see (http://www.worldatlas.com/aatlas/ctycodes.htm).
(TIF)

S6 Fig pvclust analysis of *M*. *tuberculosis* isolates belonging to the H sub-lineages. The clusters edges are numbered in grey and the AU p-values are shown in black. Strongly supported clusters with AU greater than 95% are highlighted with red rectangle. Country codes (http://www.worldatlas.com/aatlas/ctycodes.htm).
(TIF)

S7 Fig Minimum spanning tree based on spoligotypes present in Africa. Minimum spanning tree based on spoligotypes (n = 10577 isolates) showing the main SITs present in Africa. The structure of the tree is represented by links (continuous vs. dashed and dotted lines) denoting distance (changes) between patterns, and circles representing each spoligotype pattern. The size of circles is proportional to the number of isolates associated to a given SIT (SIT number in the circle (large circles) or SIT number adjacent to the circle (small circles). The figure can be zoomed for a better visualization. In the insert, the number following the lineage indicates the total number of isolates for the given lineage.
(TIF)

S8 Fig Minimum spanning tree based on LAM spoligotypes present in Africa. Minimum Spanning Trees based on spoligotypes (A) focusing on LAM sub-lineage representing n = 2719 and (B) focusing on T sub-lineages representing n = 2531 isolates. The structure of the tree is represented by links (continuous vs. dashed and dotted lines) denoting distance (changes) between patterns, and circles representing each spoligotype pattern. The size of circles is proportional to the number of isolates associated to a given SIT (SIT number in the circle (large circles) or SIT number adjacent to the circle (small circles). In the insert, the number following the sub-lineage indicates the total number of isolates for the given sub-lineage.
(TIF)

S1 Table Spoligotype data for the major *M*. *tuberculosis* lineages present in 36 countries in Africa. Spoligotype data was extracted from SITVIT2 as well as from literature for countries were spoligotype date had not been included in SITVIT2. Countries highlighted in grey were not included in the analysis as <100 *M*. *tuberculosis* isolates had been spoligotyped. Country codes (http://www.worldatlas.com/aatlas/ctycodes.htm).
(XLSX)

S2 Table Spoligotype data for the *M*. *tuberculosis* sub-lineages present in 36 countries in Africa. Spoligotype data was extracted from SITVIT2 as well as from literature for countries were spoligotype date had not been included in SITVIT2. Countries highlighted in grey were not included in the analysis as <100 *M*. *tuberculosis* isolates had been spoligotyped. Country codes (http://www.worldatlas.com/aatlas/ctycodes.htm).
(XLSX)

## References

[1] Streicher EM, Warren RM, Kewley C, Simpson J, Rastogi N, Sola C, et al Genotypic and phenotypic characterization of drug-resistant *Mycobacterium tuberculosis* isolates from rural districts of the Western Cape Province of South Africa. J Clin Microbiol. 2004; 42: 891–894. 10.1128/JCM.42.2.891-894.2004 14766882 PMC344460

[2] Filliol I, Driscoll JR, van Soolingen D, Kreiswirth BN, Kremer K, Valétudie G, et al Global distribution of *Mycobacterium tuberculosis* spoligotypes. Emerg Infect Dis. 2002; 8: 1347–1349. 10.3201/eid0811.020125 12453368 PMC2738532

[3] Brudey K, Driscoll JR, Rigouts L, Prodinger WM, Gori A, Al-Hajoj SA, et al Mycobacterium tuberculosis complex genetic diversity: mining the fourth international spoligotyping database (SpolDB4) for classification, population genetics and epidemiology. BMC Microbiol. 2006; 6: 23 10.1186/1471-2180-6-23 16519816 PMC1468417

[4] Filliol I, Driscoll JR, van Soolingen D, Kreiswirth BN, Kremer K, Valétudie G, et al Snapshot of moving and expanding clones of *Mycobacterium tuberculosis* and their global distribution assessed by spoligotyping in an international study. J Clin Microbiol. 2003; 41: 1963–1970. 10.1128/JCM.41.5.1963-1970.2003 12734235 PMC154710

[5] Gagneux S, DeRiemer K, Van T, Kato-Maeda M, de Jong BC, Narayanan S, et al Variable host-pathogen compatibility in *Mycobacterium tuberculosis*. Proc Natl Acad Sci USA. 2006; 103:2869–73. Epub 2006 Feb 13. 10.1073/pnas.0511240103 16477032 PMC1413851

[6] van Embden JD, Cave MD, Crawford JT, Dale JW, Eisenach KD, Gicquel B, et al Strain identification of *Mycobacterium tuberculosis* by DNA fingerprinting: recommendations for a standardized methodology [see comments]. J Clin Microbiol. 1993; 31: 406–409. 8381814 10.1128/jcm.31.2.406-409.1993 PMC262774

[7] Supply P, Allix C, Lesjean S, Cardoso-Oelemann M, Rusch-Gerdes S, Willery E, et al Proposal for standardization of optimized mycobacterial interspersed repetitive unit-variable-number tandem repeat typing of *Mycobacterium tuberculosis*. J Clin Microbiol. 2006; 44: 4498–4510. 10.1128/JCM.01392-06 17005759 PMC1698431

[8] Kamerbeek J, Schouls L, Kolk A, van Agterveld M, van Soolingen D, Kuijper S, et al Simultaneous detection and strain differentiation of *Mycobacterium tuberculosis* for diagnosis and epidemiology. J Clin Microbiol. 1997; 35: 907–914. 9157152 10.1128/jcm.35.4.907-914.1997 PMC229700

[9] Small PM, Hopewell PC, Singh SP, Paz A, Parsonnet J, Ruston DC, et al The epidemiology of tuberculosis in San Francisco. A population-based study using conventional and molecular methods. N Engl J Med. 1994; 330: 1703–1709. 10.1056/NEJM199406163302402 7910661

[10] Bifani PJ, Plikaytis BB, Kapur V, Stockbauer K, Pan X, Lutfey ML, et al Origin and interstate spread of a New York City multidrug-resistant *Mycobacterium tuberculosis* clone family. JAMA 1996; 275: 452–457. 8627966

[11] Gandhi NR, Moll A, Sturm AW, Pawinski R, Govender T, Lalloo U, et al Extensively Drug Resistant Tuberculosis as a cause of death in patients co-infected with Tuberculosis and HIV in a rural area of South Africa. Lancet. 2006; 368: 1575–1580. 10.1016/S0140-6736(06)69573-1 17084757

[12] Chihota VN, Muller B, Mlambo CK, Pillay M, Tait M, Streicher EM, et al Population structure of multi- and extensively drug-resistant *Mycobacterium tuberculosis* strains in South Africa. J Clin Microbiol. 2012; 50: 995–1002. 10.1128/JCM.05832-11 22170931 PMC3295122

[13] Yeh RW, Hopewell PC, Daley CL Simultaneous infection with two strains of Mycobacterium tuberculosis identified by restriction fragment length polymorphism analysis. Int J Tuberc Lung Dis. 1999; 3: 537–539. 10383069

[14] Warren RM, Victor TC, Streicher EM, Richardson M, Beyers N, Gey van Pittius NC, et al Patients with active tuberculosis often have different strains in the same sputum specimen. AmJRespirCrit Care Med. 2004;169: 610–614. 10.1164/rccm.200305-714OC 14701710

[15] Sola C, Filliol I, Gutierrez MC, Mokrousov I, Vincent V, Rastogi N. Spoligotype database of *Mycobacterium tuberculosis*: biogeographic distribution of shared types and epidemiologic and phylogenetic perspectives. Emerg Infect Dis 2001; 7: 390–396. 10.3201/eid0703.010304 11384514 PMC2631784

[16] Demay C, Liens B, Burguiere T, Hill V, Couvin D, Millet J, et al SITVITWEB—a publicly available international multimarker database for studying *Mycobacterium tuberculosis* genetic diversity and molecular epidemiology. Infect Genet Evol. 2012; 12: 755–766. 10.1016/j.meegid.2012.02.004 22365971

[17] Streicher EM, Victor TC, van der SG, Sola C, Rastogi N, van Helden PD, et al Spoligotype signatures in the *Mycobacterium tuberculosis* complex. J Clin Microbiol. 2007; 45: 237–240. 10.1128/JCM.01429-06 17065260 PMC1828946

[18] Filliol I, Driscoll JR, Van Soolingen D, Kreiswirth BN, Kremer K, Valétudie G, et al Global distribution of Mycobacterium tuberculosis spoligotypes. Emerg Infect Dis. 2002;8:1347–9. 10.3201/eid0811.020125 12453368 PMC2738532

[19] Brudey K, Filliol I, Ferdinand S, Guernier V, Duval P, Maubert B, et al Long-term population-based genotyping study of *Mycobacterium tuberculosis* complex isolates in the French departments of the Americas. J Clin Microbiol. 2006; 44: 183–191. 10.1128/JCM.44.1.183-191.2006 16390968 PMC1351934

[20] Hill V, Zozio T, Sadikalay S, Viegas S, Streit E, Kallenius G, et al MLVA based classification of *Mycobacterium tuberculosis* complex lineages for a robust phylogeographic snapshot of its worldwide molecular diversity. PLoS One. 2012; 7: e41991 10.1371/journal.pone.0041991 22984400 PMC3439451

[21] Mbugi EV, Katale BZ, Streicher EM, Keyyu JD, Kendall SL, Dockrell HM, et al Mapping of *Mycobacterium tuberculosis* Complex Genetic Diversity Profiles in Tanzania and Other African Countries. PLoS One. 2016; 11: e0154571 10.1371/journal.pone.0154571 27149626 PMC4858144

[22] Gehre F, Kumar S, Kendall L, Ejo M, Secka O, Ofori-Anyinam B, et al A Mycobacterial Perspective on Tuberculosis in West Africa: Significant Geographical Variation of *M*. *africanum* and Other *M*. *tuberculosis* Complex Lineages. PLoS Negl Trop Dis. 2016; 10: e0004408 10.1371/journal.pntd.0004408 26964059 PMC4786107

[23] Firdessa R, Berg S, Hailu E, Schelling E, Gumi B, Erenso G, et al Mycobacterial lineages causing pulmonary and extrapulmonary tuberculosis, Ethiopia. Emerg Infect Dis. 2013; 19: 460–463. 10.3201/eid1903.120256 23622814 PMC3647644

[24] Comas I, Coscolla M, Luo T, Borrell S, Holt KE, Erenso G, et al Out-of-Africa migration and Neolithic coexpansion of *Mycobacterium tuberculosis* with modern humans. Nat Genet. 2013; 45: 1176–1182. 10.1038/ng.2744 23995134 PMC3800747

[25] Hirsh AE, Tsolaki AG, DeRiemer K, Feldman MW, Small PM Stable association between strains of *Mycobacterium tuberculosis* and their human host populations. Proc Natl Acad Sci USA. 2004; 101: 4871–4876. 10.1073/pnas.0305627101 15041743 PMC387341

[26] Wirth T, Hildebrand F, lix-Beguec C, Wolbeling F, Kubica T, Kremer K, et al Origin, spread and demography of the *Mycobacterium tuberculosis* complex. PLoS Pathog. 2008; 4: e1000160 10.1371/journal.ppat.1000160 18802459 PMC2528947

[27] Couvin D, Rastogi N. The establishment of databases on circulating genotypes of *Mycobacterium tuberculosis* complex and web tools for an effective response to better monitor, understand and control the tuberculosis epidemic worldwide. Euro Reference—Journal of Reference. 2014; 12: 36–48.

[28] Couvin D, Rastogi N. Tuberculosis—A global emergency: Tools and methods to monitor, understand, and control the epidemic with specific example of the Beijing lineage. Tuberculosis. 2015; 95 Suppl 1: S177–S189. 25797613 10.1016/j.tube.2015.02.023

[29] Godreuil S, Torrea G, Terru D, Chevenet F, Diagbouga S, Supply P, et al First molecular epidemiology study of *Mycobacterium tuberculosis* in Burkina Faso. J Clin Microbiol. 2007; 45: 921–927. 10.1128/JCM.01918-06 17251410 PMC1829100

[30] Homolka S, Post E, Oberhauser B, George AG, Westman L, Dafae F, et al High genetic diversity among *Mycobacterium tuberculosis* complex strains from Sierra Leone. BMC Microbiol. 2008; 8: 103 10.1186/1471-2180-8-103 18578864 PMC2447842

[31] Affolabi D, Anyo G, Faihun F, Sanoussi N, Shamputa IC, Rigouts L, et al First molecular epidemiological study of tuberculosis in Benin. Int J Tuberc Lung Dis. 2009; 13: 317–322. 19275790

[32] Yeboah-Manu D, sante-Poku A, Bodmer T, Stucki D, Koram K, Bonsu F, et al Genotypic diversity and drug susceptibility patterns among *M*. *tuberculosis* complex isolates from South-Western Ghana. PLoS One; 2011; 6: e21906 10.1371/journal.pone.0021906 21779354 PMC3133566

[33] Blouin Y, Hauck Y, Soler C, Fabre M, Vong R, Dehan C, et al Significance of the identification in the Horn of Africa of an exceptionally deep branching *Mycobacterium tuberculosis* clade. PLoS One. 2012; 7: e52841 10.1371/journal.pone.0052841 23300794 PMC3531362

[34] Gafirita J, Umubyeyi AN, Asiimwe BB A first insight into the genotypic diversity of *Mycobacterium tuberculosis* from Rwanda. BMC Clin Pathol. 2012; 12: 20 10.1186/1472-6890-12-20 23131092 PMC3520741

[35] Ouassa T, Borroni E, Loukou GY, Faye-Kette H, Kouakou J, Menan H, et al High prevalence of shared international type 53 among *Mycobacterium tuberculosis* complex strains in retreated patients from Cote d'Ivoire. PLoS One. 2012; 7: e45363 10.1371/journal.pone.0045363 23028962 PMC3445461

[36] Traore B, Diarra B, Dembele BP, Somboro AM, Hammond AS, Siddiqui S, et al Molecular strain typing of *Mycobacterium tuberculosis* complex in Bamako, Mali. Int JTubercLung Dis. 2012; 16: 911–916. 10.5588/ijtld.11.0397 22508197

[37] Ejo M, Gehre F, Barry MD, Sow O, Bah NM, Camara M, et al First insights into circulating *Mycobacterium tuberculosis* complex lineages and drug resistance in Guinea. Infect Genet Evol. 2015; 33: 314–319. 10.1016/j.meegid.2015.05.022 26004194 PMC4503999

[38] Lukoye D, Katabazi FA, Musisi K, Kateete DP, Asiimwe BB, Okee M, et al The T2 *Mycobacterium tuberculosis* genotype, predominant in Kampala, Uganda, shows negative correlation with antituberculosis drug resistance. AntimicrobAgents Chemother. 2014; 58: 3853–3859. 10.1128/AAC.02338-13 PMC4068514 24777100

[39] Perdigao J, Clemente S, Ramos J, Masakidi P, Machado D, Ramos J, et al Genetic diversity, transmission dynamics and drug resistance of *Mycobacterium tuberculosis* in Angola. Sci Rep. 2017; 7: 42814 10.1038/srep42814 28230095 PMC5322374

[40] Team RC A language and environment for statistical computing. R Foundation for Statistical Computing, Vienna, Austria. 2015. Available from: http://wwwR-projectorg/:

[41] Q.Vu V. ggbiplot: A ggplot2 based biplot. 2011. Available from: http://githubcom/vqv/ggbiplot.

[42] Suzuki R, Shimodaira H Pvclust: an R package for assessing the uncertainty in hierarchical clustering. Bioinformatics. 2006; 22: 1540–1542. 10.1093/bioinformatics/btl117 16595560

[43] Mokrousov I, Vyazovaya A, Iwamoto T, Skiba Y, Pole I, Zhdanova S, et al Latin-American-Mediterranean lineage of *Mycobacterium tuberculosis*: Human traces across pathogen's phylogeography. Mol Phylogenet Evol. 2016; 99: 133–143. 10.1016/j.ympev.2016.03.020 27001605

[44] Hanekom M, van der Spuy GD, Gey Van Pittius NC, McEvoy CR, Ndabambi SL, Victor TC, et al Evidence that the spread of *Mycobacterium tuberculosis* strains with the Beijing genotype is human population dependent. J Clin Microbiol. 2007; 45: 2263–2266. 10.1128/JCM.02354-06 17475755 PMC1933015

[45] Merker M, Blin C, Mona S, Duforet-Frebourg N, Lecher S, Willery E, et al Evolutionary history and global spread of the *Mycobacterium tuberculosis* Beijing lineage. Nat Genet. 2015; 47: 242–249. 10.1038/ng.3195 25599400 PMC11044984

[46] Salie M, van der ML, Moller M, Daya M, van der Spuy GD, van Helden PD, et al Associations between human leukocyte antigen class I variants and the *Mycobacterium tuberculosis* subtypes causing disease. J Infect Dis. 2014; 209: 216–223. 10.1093/infdis/jit443 23945374 PMC3873786

[47] Asante-Poku A, Yeboah-Manu D, Otchere ID, Aboagye SY, Stucki D, Hattendorf J et al *Mycobacterium africanum* is associated with patient ethnicity in Ghana. PLoS Negl Trop Dis. 2015; 9(1):e3370 10.1371/journal.pntd.0003370 eCollection 2015 Jan. 25569290 PMC4287525

