# Supplementary figures and images for: Geospatial distribution of Mycobacterium tuberculosis genotypes in Africa
Source: PLoS One. 2018 Aug 1;13(8):e0200632. doi: 10.1371/journal.pone.0200632 (PMC6070189; doi:10.1371/journal.pone.0200632)

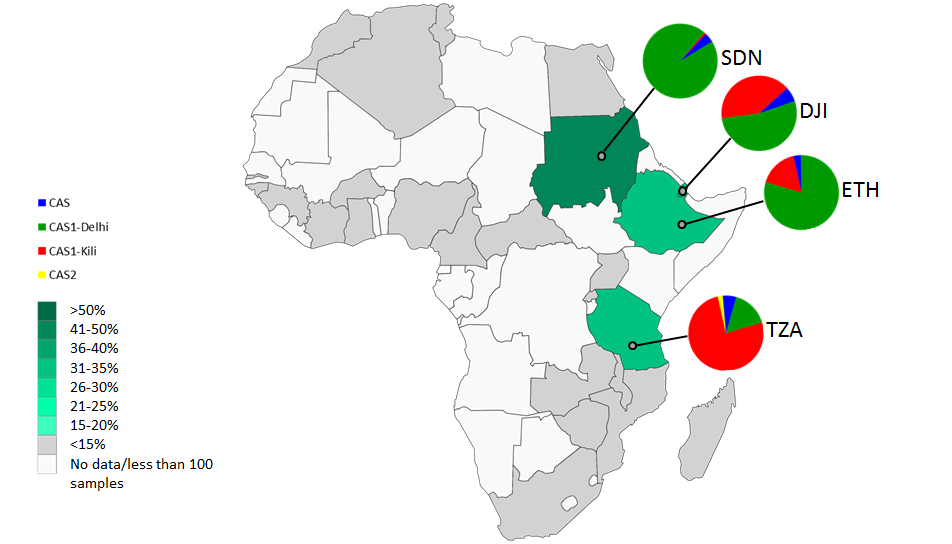

Supplement: S1 Fig — Country specific spoligotype data was only included if the country had >100 M. tuberculosis isolates and ≥15% of these isolates were from the CAS lineage. The sizes of the pie chart segments depict the proportion of isolates belonging to the different CAS sub-lineages (see colour chart for the respective sub-lineages). Each country has been shaded according to the proportion of CAS sub-lineages isolates present in that country (see colour intensity chart). Country codes (http://www.worldatlas.com/aatlas/ctycodes.htm). (TIF) [file pone.0200632.s001.tif]

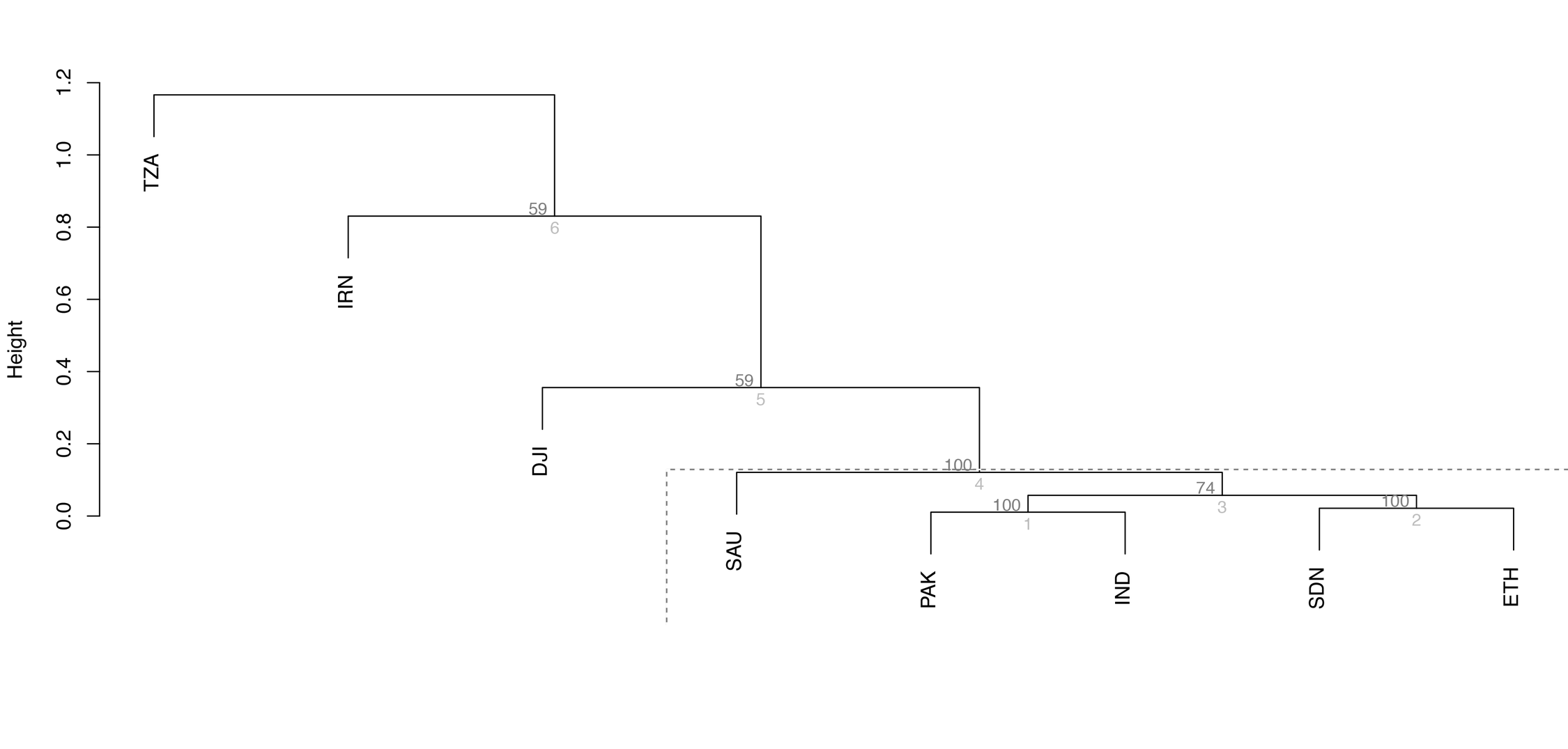

Supplement: S2 Fig — The clusters edges are numbered in grey and the AU p-values are shown in black. Strongly supported clusters with AU greater than 95% are highlighted with red rectangle. Country codes (http://www.worldatlas.com/aatlas/ctycodes.htm). (TIF) [file pone.0200632.s002.tif]

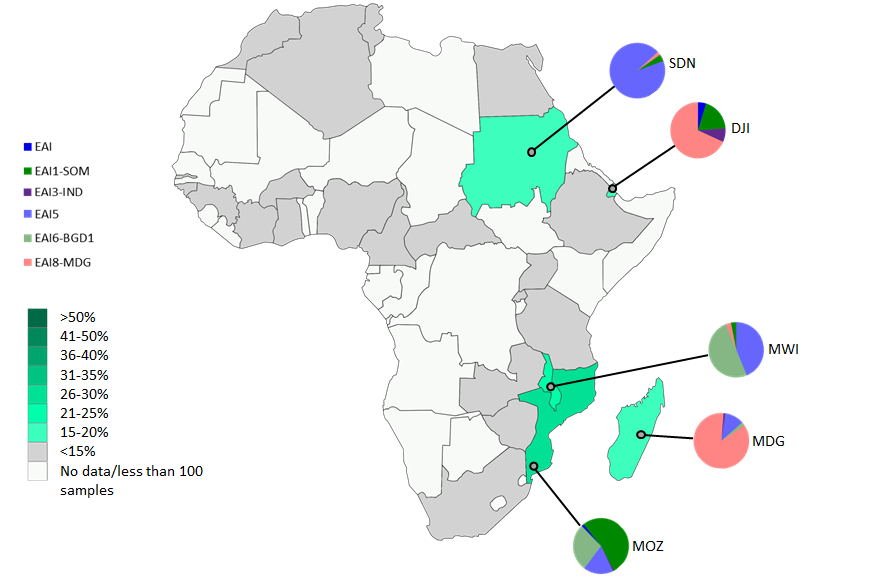

Supplement: S3 Fig — Country specific spoligotype data was only included if the country had >100 M. tuberculosis isolates and ≥15% of these isolates were from the EAI lineage. The sizes of the pie chart segments depict the proportion of isolates belonging to the different EAI sub-lineages (see colour chart for the respective sub-lineages). Each country has been shaded according to the proportion of EAI sub-lineages isolates present in that country (see colour intensity chart). Country codes (http://www.worldatlas.com/aatlas/ctycodes.htm). (TIF) [file pone.0200632.s003.tif]

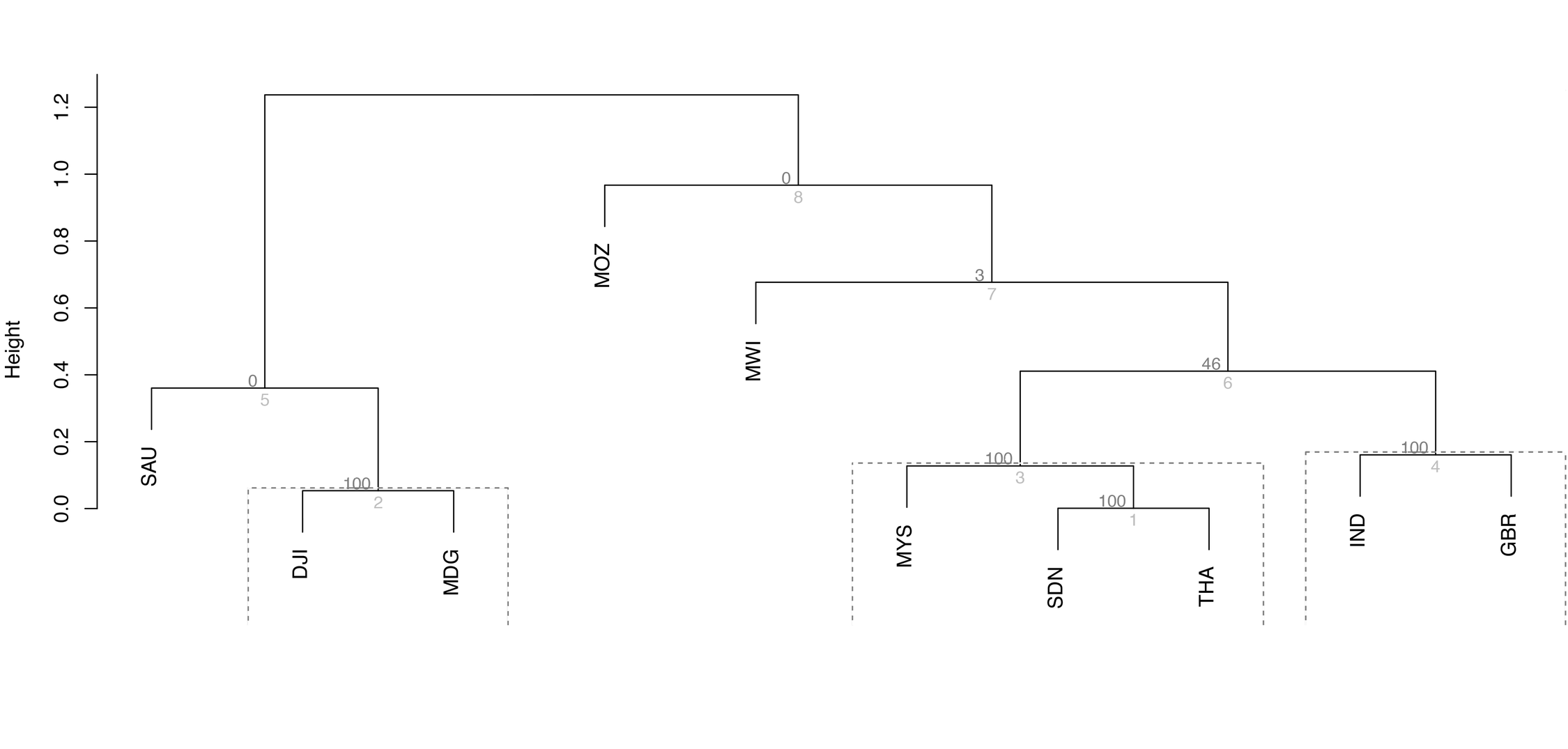

Supplement: S4 Fig — The clusters edges are numbered in grey and the AU p-values are shown in black. Strongly supported clusters with AU greater than 95% are highlighted with red rectangle. Country codes (http://www.worldatlas.com/aatlas/ctycodes.htm). (TIF) [file pone.0200632.s004.tif]

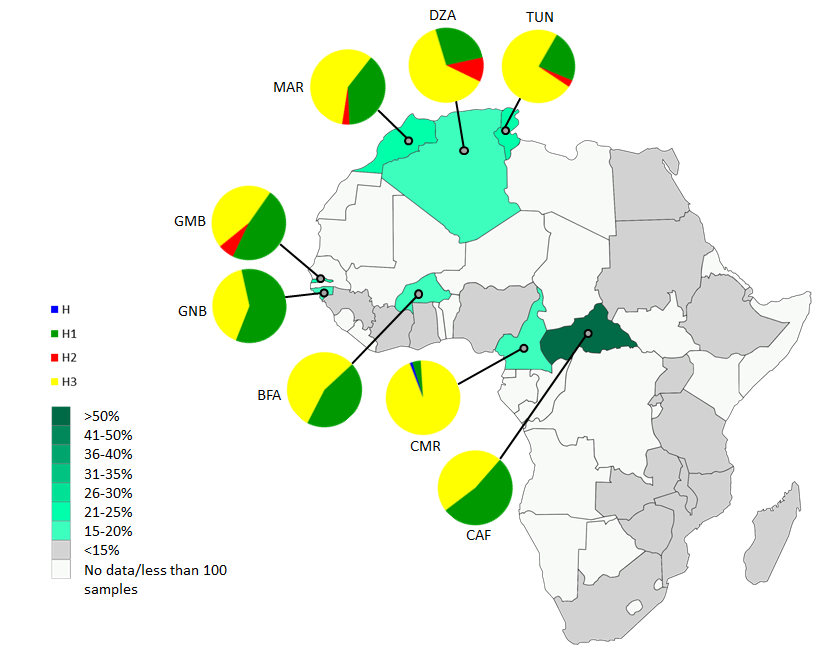

Supplement: S5 Fig — Country specific spoligotype data was only included if the country had >100 M. tuberculosis isolates and ≥15% of these isolates were from the H lineage. The sizes of the pie chart segments depict the proportion of isolates belonging to the different H sub-lineages (see colour chart for the respective sub-lineages). Each country has been shaded according to the proportion of H sub-lineages isolates present in that country (see colour intensity chart). Country codes see (http://www.worldatlas.com/aatlas/ctycodes.htm). (TIF) [file pone.0200632.s005.tif]

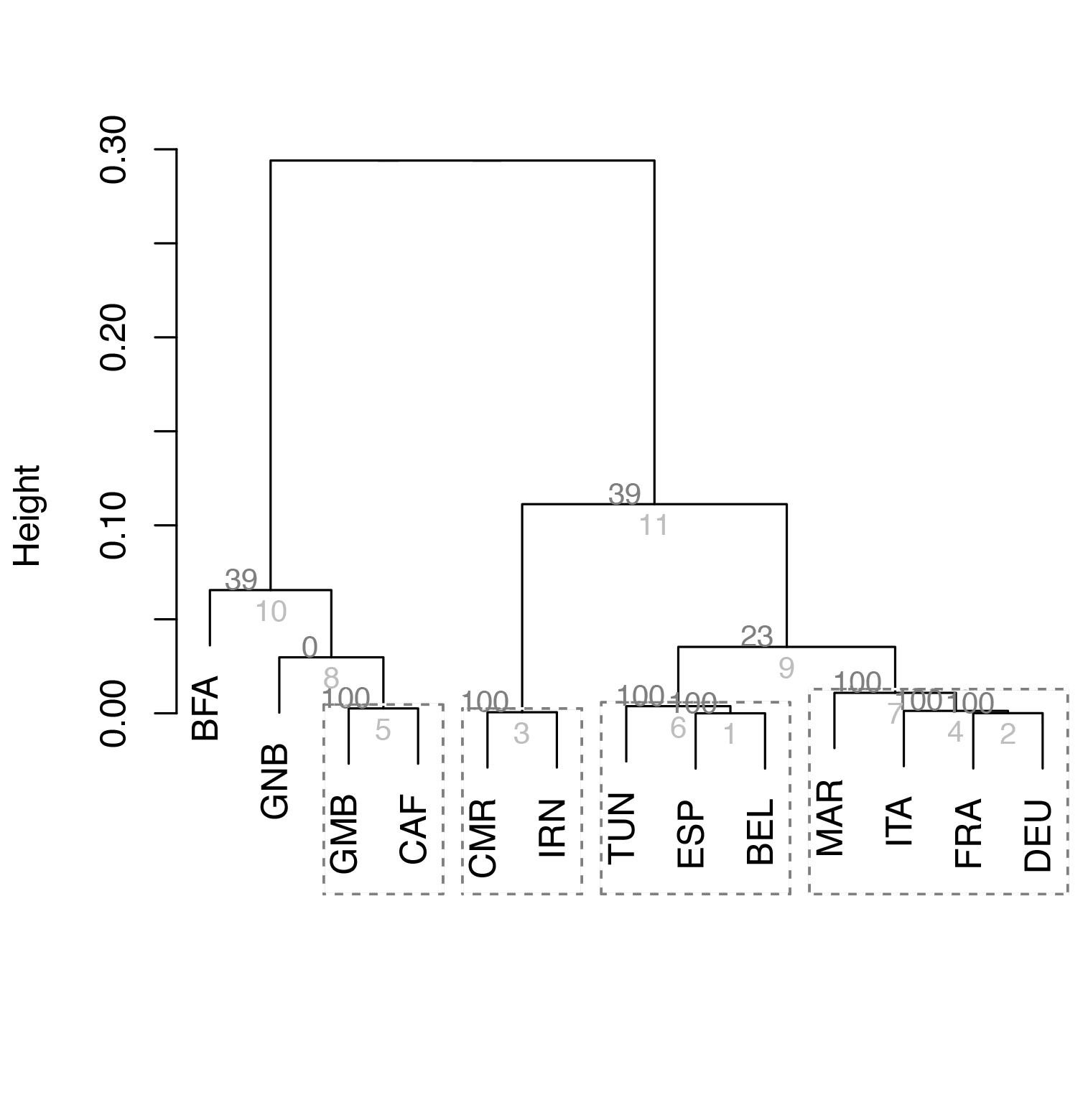

Supplement: S6 Fig — The clusters edges are numbered in grey and the AU p-values are shown in black. Strongly supported clusters with AU greater than 95% are highlighted with red rectangle. Country codes (http://www.worldatlas.com/aatlas/ctycodes.htm). (TIF) [file pone.0200632.s006.tif]

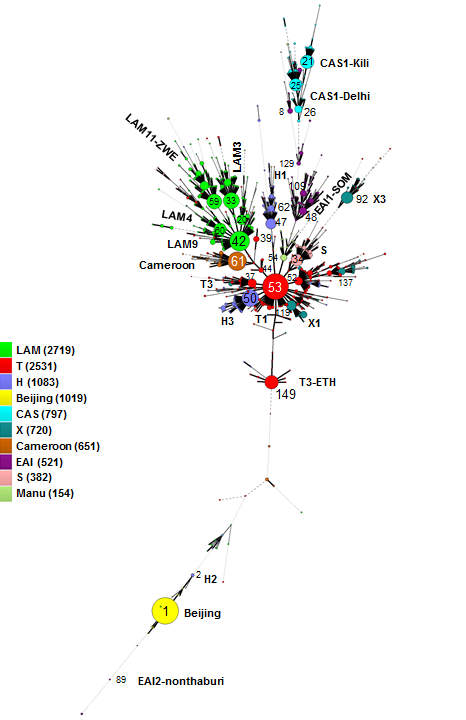

Supplement: S7 Fig — Minimum spanning tree based on spoligotypes (n = 10577 isolates) showing the main SITs present in Africa. The structure of the tree is represented by links (continuous vs. dashed and dotted lines) denoting distance (changes) between patterns, and circles representing each spoligotype pattern. The size of circles is proportional to the number of isolates associated to a given SIT (SIT number in the circle (large circles) or SIT number adjacent to the circle (small circles). The figure can be zoomed for a better visualization. In the insert, the number following the lineage indicates the total number of isolates for the given lineage. (TIF) [file pone.0200632.s007.tif]

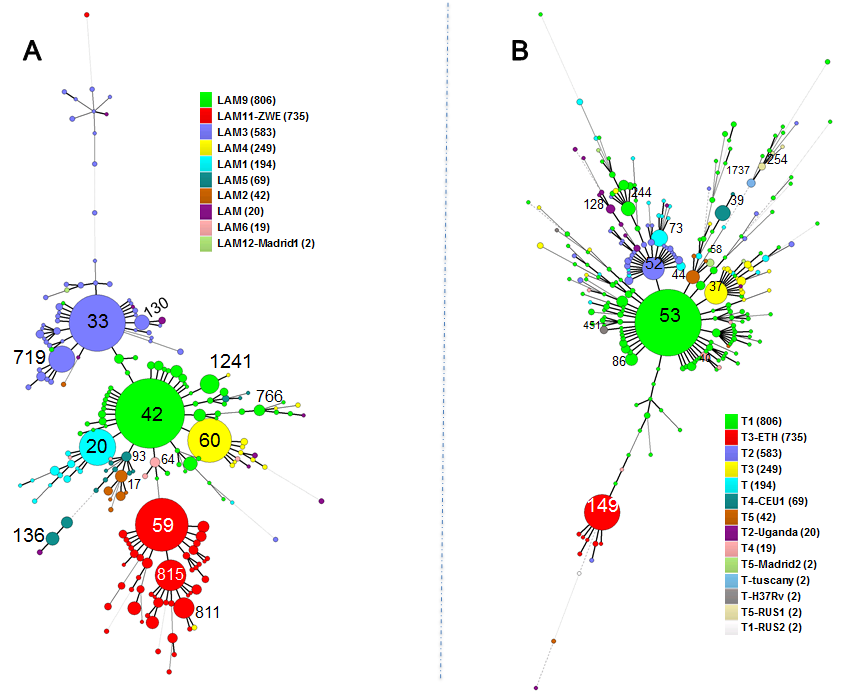

Supplement: S8 Fig — Minimum Spanning Trees based on spoligotypes (A) focusing on LAM sub-lineage representing n = 2719 and (B) focusing on T sub-lineages representing n = 2531 isolates. The structure of the tree is represented by links (continuous vs. dashed and dotted lines) denoting distance (changes) between patterns, and circles representing each spoligotype pattern. The size of circles is proportional to the number of isolates associated to a given SIT (SIT number in the circle (large circles) or SIT number adjacent to the circle (small circles). In the insert, the number following the sub-lineage indicates the total number of isolates for the given sub-lineage. (TIF) [file pone.0200632.s008.tif]
